# Supplementary material for: Are carbon emissions trading and green financial instruments synergistic? -Comprehensive quantitative research based on content analysis
Source: PLoS One. 2024 Mar 7;19(3):e0298601. doi: 10.1371/journal.pone.0298601 (PMC10919601; doi:10.1371/journal.pone.0298601)
Supplement: S1 Data — (ZIP) [file pone.0298601.s001.zip › Data/地级市Z_F工作报告.zip!/║╙─╧/╨┬╧τ╩╨/└┤╫╘╣1⁄2╓┌║┼í╛┬φ┐╦╩2╛▌═°í┐_2005.docx]

各位代表：

　　现在，我代表市人民政府向大会作工作报告，请予审议，并请各位政协委员和其他列席人员提出意见。

一、二OO四年工作回顾

　　2004年，新一届政府在省委、省政府和市委正确领导下，在市人大、市政协监督支持下，团结带领全市人民，牢固树立科学发展观，积极顺应国家宏观调控，迎难而上，开拓进取，各项工作取得新成绩，实现了本届政府的良好开局。

　　主要经济指标突破性增长，超额完成年度发展目标。预计完成生产总值461.2亿元，增长14.6%，为1997年以来最高增幅，高于本届政府任期五年预计年均增速（下同）3.6个百分点。全社会固定资产投资220亿元，增长45.6%，高于预计年均增速25.6个百分点。限额以上工业企业增加值124亿元，增长22.2%，高于预计年均增速9.2个百分点。地方财政一般预算收入19.9亿元，增长27.4%，高于预计年均增速16.4个百分点。社会消费品零售总额156亿元，增长17.1%，高于预计年均增速7.1个百分点。粮食总产295.5万吨，增长5.3%。工业经济五大强势产业和七大特色产业销售收入分别增长32.4%、25.5%。中心城市和县域经济竞相发展两大格局基本形成。被国家统计局评定为全国综合实力百强市。

　　调整优化经济结构，发展活力进一步增强。实施粮食优质化工程，优质小麦播种面积325万亩，占麦播总面积的72%，新良、长远公司成为国家级龙头企业。畜牧业产值占农业总产值比重达到40.2%，提高2.6个百分点；养殖小区达到150个，奶类产量增长42%。超额完成植树造林年度任务。农村沼气入户率15%以上。以发展规模经济和资源节约型经济为主导，关闭淘汰污染严重、资源浪费企业33家，对以11家省重点企业为核心的50家重点企业进行扶持，50家重点企业销售收入201.5亿元，占限额以上工业比重达到52.9%。高新技术产业增加值38亿元，占全部工业增加值比重达到24%，提高1个百分点。民营经济增加值245.3亿元，占全市GDP的53%，提高3个百分点。县域GDP总量达到315.5亿元，占全市GDP的68.2%，提高1.8个百分点。城镇化率达到32 %，提高1.8个百分点，唐庄、孟庄等8个镇升级为国家级重点镇。全市金融机构各项存、贷款余额分别比年初增长12.2%和8.7%。全社会用电量增长18.2%，其中工业用电量增长19.7%。旅游门票收入增长78.7%。财产和人寿保险收入分别增长8%和42.6%。

　　固定资产投资结构更趋合理，重点项目建设进展顺利。工业投资94.2亿元，增长81.6%，占城镇以上固定资产投资的50.8%。民间投资89亿元，增长110.5%，占城镇以上固定资产投资的48%。实施千万元以上项目380个，增加90个，当年完成投资153.1亿元。其中118项重点项目总投资475亿元，当年完成投资83.9亿元，续建新建项目中孟电集团日产2×3000吨干法水泥熟料、金龙铜业外翅片强化传热铜管、中新化工年产10万吨联碱等13个项目竣工投产；白鹭化纤年产万吨差别化新型连续纺粘胶长丝和6000吨差别化氨纶、赵固煤矿年产240万吨原煤等34个项目顺利开工。

　　基础设施建设步伐加快，城乡面貌有较大改观。完成行政中心东迁，城市功能分区更趋合理。新修改造牧野路等16条路段，整治街巷28条。小尚庄污水处理厂和垃圾无害化处理场建设顺利。城市亮化工程成效明显。调整、新增公交线路15条，更新车辆78部，群众出行更加方便。牧野公园改造一期工程建成开放。新建街头小游园30个。新增绿地面积200万平方米。商品房竣工面积48万平方米。城区气化率80%，提高12个百分点。城乡电网建设改造投资6660万元。完成公路投资16.7亿元，新增干线公路67公里、县乡公路132公里和“村村通”行政村56个。市区至凤泉区、获嘉、原阳、小店工业区4条快速路主体工程完工。京珠高速新乡段、新庄―原阳高速公路建成通车，济东高速、阿深高速新乡段开工建设。

　　各项改革稳步推进，对外开放取得新成绩。市县政府机构改革任务顺利完成。事业单位体制改革稳妥推进，撤销及退出事业机构序列单位29家。列入省改制范围的156家国有（集体）企业完成或基本完成改制的93家，改制面59.7%，其中市属83家企业已完成产权制度改革的18家，即将进行实质性操作的44家。超额完成国有粮食购销企业改革人员分流任务。农村信用社改革清产核资、增资扩股全面完成，清收不良贷款1.79亿元。农业税减免和对种粮农民直接补贴足额到位。华兰生物成功上市。引进江苏太平洋建设有限公司参与城市基础设施建设;有偿转让出租车经营权和拍卖城市道路冠名权，筹集资金3632万元。上海华源、上海希纳、福建华亚、陕西石羊、浙江万向、江苏金谷、北京建谊、广东香江等一批国内知名企业在新乡投资发展。引进市外资金30.1亿元，增长31.9%。实际利用外资4563万美元,增长0.8%；外贸进出口总值3.6亿美元，增长37.9%。

　　实施科教兴市、人才强市战略，各项社会事业全面发展。着力增强经济发展的智力支持和人才支撑，科技创新能力不断提高。市农科院两项育种研究课题列入国家高技术“863”计划子项目。8家企业被认定为省高新技术企业，74个项目被列入国家和省科技发展计划，获省科技进步奖7项，居全省第3位。组织申报专利700余项，居全省第2位。教育资源进一步整合优化，优质教育规模扩大，农村中小学库内D级危房改造基本完成。支持驻新高校发展，完成征地624.5亩。新型农村合作医疗参合率77.6%，226.8万农民得到医疗保障。新建改造中心乡镇卫生院39所。水、大气等污染治理取得新成效。新建高标准社区服务中心20个，社区服务业加盟企业发展到100家。加强计划生育基层基础工作，人口自然增长率控制在5.1‰以内。完成市区地名规划。成功举办市第八届运动会。新闻出版、广播电视、审计、统计、气象、防震减灾、外事、侨务、地方史志、档案、民族宗教、人防等各项事业都取得了新成绩。

　　加快完善社会保障体系，人民生活水平稳步提高。提高养老保险市级统筹水平，依法加大社会保险征缴清欠力度，养老保险金、下岗职工基本生活费、失业保险金发放率均为100%。全市新增就业6.7万人，安置下岗失业人员4.7万人，其中“4050”人员1.3万人。12.9万人享受城市低保，基本实现了动态管理下的应保尽保。728名特困患者得到医疗救助，19184名贫困学生得到资助和减免学费，2.9万人实现脱贫。城镇居民人均可支配收入7146元，增长14.7%；农民人均纯收入2748元，增长14.1%。

　　推进政治文明和精神文明建设，保持社会大局稳定。认真执行市人大及其常委会通过的决定和决议，积极组织政府职能部门向人大述职，自觉接受政协民主监督，广泛听取社会各界意见和建议。深入开展“五城”联创活动，市民文明素质进一步提高。“信用新乡”建设成效明显，被中宣部定为全国信用建设试点市。《牧野文化丛书》编撰工作进展顺利。广泛开展国防教育，军政、军民关系更加密切。廉政建设和反腐败斗争不断深入。严格落实安全生产工作责任制，大力开展专项整治，安全生产形势平稳。积极创建“安全城市”，加强社会治安综合治理，开展严打整治斗争，集中处理信访突出问题及群体性事件，社会大局稳定。

　　努力建设服务型政府，发展环境不断优化。认真贯彻落实《行政许可法》，健全完善行政服务中心功能，全面推行许可事项听证、公示制度，建立新闻发言人制度，扩大政务公开，促进决策的科学化、民主化。建立行政首长问责制，完善行政效能监察制度，各级政府工作人员责任意识、服务意识不断增强。依法查处“四乱”案件，对医用卫材、食品等行业进行专项整治，建立政府、部门和周边村庄三位一体企业服务机制，经济发展环境进一步改善。

　　各位代表，2004年在国家加强宏观调控的大背景下，我市经济在新一轮发展中抓住了机遇，加快了发展，取得了新成绩，这主要归功于省委、省政府和市委的正确领导，归功于市人大、市政协的监督和支持，归功于全市上下形成的加快发展的责任意识、奋发有为的进取精神和真抓实干的工作作风。在此，我谨代表市人民政府，向各位人大代表和政协委员，向广大工人、农民、知识分子、干部、驻新解放军指战员、武警官兵、公安干警及社会各界人士，向关心、支持新乡建设的香港特别行政区同胞、澳门特别行政区同胞、台湾同胞、海外侨胞和国际友人致以崇高敬意和衷心感谢！

　　回顾全年工作，必须清醒地看到，经济社会发展还存在诸多困难和问题，我市经济快速增长的基础还不稳固，工业企业成长的动力和活力不足，经济社会发展面临的竞争局面日趋严峻。一是整体经济实力不强，第二产业比重不高，工业经济结构性矛盾突出。预计2004年我市人均GDP、人均工业增加值和人均财政收入均列全省9位左右；第二产业比重仅为50.3%，列全省第12位。二是县域经济发展不快，特色产业不突出，农业产业化水平不高，农民增收靠天、靠政策的因素较多，稳定增收的基础不牢固。三是财政收入规模偏小，收支矛盾突出，市级财政调控能力减弱，压力增大。四是国企改革任务重，困难大，成本过高。五是就业再就业压力持续加大，社会保障任务艰巨。每年全市约需安置新增下岗失业人员、城镇劳动力和失地农民10万多人；养老保险基金缺口大，欠缴严重，提高养老保险金标准后，今年缺口将高达8873万元。六是由于阶段性和历史性遗留问题引发的群体性突发事件时有发生，信访稳定工作压力较大。七是一些干部工作标准低、作风不扎实，不敢触及矛盾，不敢承担责任，在抓工作落实上缺乏拼劲、韧劲和创新精神，不正之风和铺张浪费现象还不同程度存在等等。这些问题需要我们认真加以解决。

二、二OO五年政府工作的总体要求　预期目标和主要措施

　　2005年是 “十五”计划的最后一年，做好今年各项工作，保持经济社会健康发展，对高质量完成“十五”计划，奠定“十一五”良好发展基础，全面建设小康社会具有重要意义。分析我市发展面临的形势，既有新的机遇，也存在困难与挑战。一方面影响经济平稳快速增长的体制性、机制性和结构性矛盾依然存在，资金、土地两大生产要素约束和煤电油运紧张局面短期内难以根本缓解。另一方面中央提出坚持科学发展观，落实“五个统筹”，加强经济社会薄弱环节，尤其是出台扶持粮食主产区、促进中部崛起等政策，有利于我市改善和提升经济社会发展的基础条件。“入世”过渡期结束以及国际国内产业大规模转移，有利于我市充分利用两个市场、两种资源，承接发达地区产业转移。特别是近几年来，我市审时度势，坚持以项目统领经济工作全局，推进经济结构调整升级，扩大固定资产投资规模，举全市之力招商引资，经济增长的内生动力正逐步增强。只要我们切实增强责任感、紧迫感，牢牢抓住机遇，主动适应调整，最大限度利用有利条件，奋发有为，扎实工作，就一定能够实现更快更好的发展。

　　今年政府工作的指导思想是：以邓小平理论和“三个代表”重要思想为指导，认真贯彻党的十六大及十六届三中、四中全会和中央经济工作会议、省委工作会议精神，坚持科学发展观，以调整结构和提高效益为主导，抓住国家促进中部崛起的历史机遇，加快改革开放，强化项目带动，发展循环经济，全力推进工业化、城镇化、农业现代化和服务业现代化进程，建设中原地区先进加工业基地、高素质人力资源培育基地和市场物流中心、现代农业示范中心，以人为本，统筹发展各项社会事业，构建和谐文明社会。

　　我市经济社会发展的预期目标是: 生产总值增长12%以上，其中三次产业分别增长5%、15%、10%；全社会固定资产投资增长25%左右；社会消费品零售总额增长11%；地方财政一般预算收入增长14%；利用市外资金增长25%；实际利用外资增长25%；外贸出口增长15%；城镇居民人均可支配收入增长10%，农民人均纯收入增长5%；城镇新增就业5.5万人；城镇登记失业率控制在4.5%以内；人口自然增长率控制在5.9‰以内。

　　市政府将按照工作总体要求，在全面完成经济社会发展任务的同时，切实办好与群众生活紧密相关的10件实事：1、确保免征农业税、对种粮农民直接补贴政策落实到位；2、实施“引石济卫”，基本实现卫河、人民胜利渠水体变清；3、完成卫河公园改造，确保牧野广场二期、科技文化公园建成开园；4、完成市疾病预防控制中心、中心医院外科病房楼、人民医院综合病房楼、传染病应急医疗救治病房楼和新区公疗医院等项目建设，改造14所中心乡镇卫生院和99所乡镇卫生院；5、落实好提高企业离退休职工养老金标准政策；6、新建9万平方米经济适用房，新增享受廉租房补助100 户；7、按照国家规定标准发放编制内教师工资，逐步提高基层干部工资待遇；8、完成老年活动中心、老年大学综合楼建设；9、解决好重污染地区1.4万人的饮水安全和山丘区1.5万人的饮水困难问题，完成山区移民扶贫720人；10、关闭不能稳定达标的制浆造纸企业和58家水泥企业中环保不达标的立窑生产线，促进水、大气环境质量进一步好转。

　　今年要重点抓好以下十个方面工作：

　　（一）壮大县域经济，推进农业和农村经济结构调整

　　增强县域经济竞争力和综合实力。加大对县域经济发展的支持力度，落实激励支持县域经济发展的各项优惠政策，发挥奖励资金作用，鼓励各县（市）发挥比较优势，发展特色经济和非公有制经济，培育支柱产业。依托重点城镇、特色工业园区、高效农业园区和农贸市场，以辉县市孟庄、新乡县三镇一区、原阳县桥北新镇、长垣县魏庄等为示范，带动农村区域经济中心发展。进一步完善民营经济发展规划和特色产业发展规划，重点发展7个特色产业和15家产值超亿元的龙头企业，培育一批产值超5亿元的大型龙头企业。大力发展农村第三产业，加大劳务输出工作力度，年内劳务输出65万人以上。

　　加大农业结构调整力度。做大做强粮食产业，培育优势粮食品牌，提高粮食加工转化能力，争取实施优质小麦生产基地“填平补齐”项目。建立完善畜禽疾病防治、良种繁育、饲料生产、产品加工和市场信息等五大体系，积极推进沿黄绿色养殖带和沿京广线猪禽生产基地建设，重点扶持10家畜牧业加工企业和40个养殖小区。实施无公害农产品行动计划，新建基地20万亩，认证产品20个以上；无公害畜产品产地认定企业达到60家，产品认证企业达到5家。逐步在市区实行蔬菜、水果、畜禽和水产品市场准入制度。完善黄河护堤林和城市生态防护林工程，实现全市平原绿化高级达标。提高农业产业化经营水平，培育壮大新良粮油、长远集团、五得利面粉、金粒麦业、敦煌新科种业、金利尔、豫牛、绿源等农牧业产业化龙头企业。新发展农民专业合作经济组织30个。

　　增强农业综合生产能力。坚持扶持“三农”力度不减弱，积极争取中央、省各项支农政策支持，增加重大农业技术推广、良种和农机具购置专项补贴。继续实施天然文岩渠治理、正面水库除险加固等防洪除涝工程和大功、韩董庄配套等节水灌溉工程，做好南水北调工程开工准备，推进三郊口水库除险加固、地下水保护行动计划等项目申报争取工作。实施山区移民扶贫和黄河滩区灾民移民迁建。抓好农业综合开发，提高农业科技和机械装备水平。继续推进生态家园富民工程，新发展农村沼气用户8万户。

　　（二）围绕提升工业经济竞争力，加大项目实施和重点企业培育力度

　　树立循环经济理念，推行资源节约与综合利用，依靠科技进步和技术创新，着力培育重点企业集团，发展壮大五大强势产业和七大特色产业，促进产业集聚发展，加快工业化进程。

　　做大做强重点企业。落实各项优惠政策，集中政府可调控资源，争取省里政策支持，扶持50家重点企业提速发展。支持优势企业建立产业园区，实施多元化经营和低成本扩张，迅速做大做强。新飞电器、金龙铜业年销售收入确保超过30亿元；白鹭化纤、安美公司、环宇电源、新亚集团、新航集团年销售收入力争达到20亿元；新乡火电厂、华星药厂、飘安集团年销售收入突破10亿元。

　　培育提升五大强势产业和七大特色产业。按照“面向市场、发挥优势、突出重点、强化投入、壮大产业”的原则，重点做好高新技术的研究开发、基地建设、企业和产品的培育、服务体系建设、人才队伍建设等工作，促进产业快速发展。五大强势产业、七大特色产业销售收入分别增长22%和23%。

　　加大项目实施力度，促进产业集聚发展。计划实施投资千万元以上项目426 项，总投资622亿元，年度投资225亿元。其中重点项目138项，总投资510.6亿元，年度投资122.1亿元。加大金龙铜业年加工6万吨精密铜管、宝泉4×30万千瓦抽水蓄能电站、豫新电厂2×30万千瓦热电联产机组等88个续建项目投资强度，完成投资99.2亿元。确保新亚集团年产10万吨杨木浆和15万吨白卡纸、新航集团年产1130万只内燃机旋装式滤清器、平原和天瑞公司日产5000吨新型干法水泥等40个项目建成投产。开工建设新飞税控收款机及网络计算机、宝山电厂2×60万千瓦机组、杭州娃哈哈和新加坡米恩公司年产4亿袋方便面、万向制动器公司年产100万只汽车制动器等50个项目，完成投资22.9亿元。积极谋划和争取实施新亚集团年产10万吨杨木化机浆20 万吨轻量涂布纸、世纪新峰公司日产万吨水泥熟料等65个投资113亿元的项目。加快高新技术开发区、小店工业区、汽车及零部件工业园和各类特色园区建设，尽快形成产业集群。

　　强化对工业经济运行的协调服务。继续加强煤电油运等生产要素的协调，保证重点企业、重点项目需要。加强金企沟通，探索建立由政府、银行、金融监管部门组成的金融稳定协调机制。加大进口设备免税与国产设备抵免所得税工作力度，降低企业成本。加快标准厂房建设，促使工业集约发展，探索建立严格土地管理制度下的工业快速发展机制。

　　（三）加快城市和交通基础设施建设，推进城镇化进程

　　坚持规划先行，做好新乡市城市总体规划、市域城镇体系规划修编，完成市区部分区域和城中村改造详细规划。加强规划管理，依法查处违法建设。统一规划建设道路、供排水、热网等基础设施，提高城市关联和资源共享程度，逐步构筑“一城三区五星环绕和两个副中心” （市区为中心，风泉、小店、小冀为支点，卫辉、辉县、获嘉、原阳、延津县城为卫星城，长垣、桥北新镇为两个副中心）的发展布局，形成中心城市和县域经济竞相发展的两大格局。

　　以城建交通六大工程为载体，加快城乡基础设施建设。实施路网工程。强力推进济东、阿深高速公路新乡段建设，做好新晋高速前期工作；加快26条588公里干线公路、环城路、县乡公路等续建项目建设，新开工21条755公里干线公路、快速路、县乡公路和9个市（县）客货场站项目。新增“村村通”行政村200个；新建、改造、续建15条24.7公里城市道路。实施环保工程。完成小尚庄污水处理厂、东孟姜女河和赵定排泵站及生活垃圾无害化处理场一期工程，开工建设市医疗垃圾处理场；争取多开工建设县城污水和垃圾处理项目；投资1.6亿元，敷设供热管网10公里。实施河渠工程。完成卫河、西孟姜女河、赵定排、人民胜利渠和共产主义渠年度整治任务。实施园林绿化工程。抓好新建道路绿化，建设一批小游园。实施大型公共设施工程。推进火车站广场改造一期、时代广场等商业设施项目建设。建成与公务员小区配套的学校、医院、商业网点等生活服务设施。启动新区行政大楼周边公共服务项目建设。实施住宅工程。房地产开发100万平方米以上，竣工50万平方米，完成投资10亿元。公务员小区年内交付使用。

　　推进以城市基础设施产权制度改革为核心的城镇化配套政策落实。重点做好公交、供水、园林绿化、污水和垃圾处理等公用事业体制改革，促进城镇化建设主体多元化。实行“土地储备中心牵头，多方联合，收益分成”的旧城改造运作机制。完善鼓励农民进城、实现农村土地资源有效整合的政策体系和城镇养老、失业、医疗等社会保障体系，加快 “城中村”改造和“村改居”步伐。城镇化率提高1.7个百分点。

　　（四）以发展现代服务业和建设物流项目为重点，加快区域商贸物流中心建设

　　全面实施市场体系规划纲要，运用先进技术和现代流通方式，进一步壮大提升商贸流通业、房地产与市政服务业、金融保险业、教育卫生服务业四个传统行业，继续大力发展现代物流业、信息服务业、文化旅游业、中介服务业、社区服务业五个新兴行业。完善以行业协会为主体的中介服务体系，着力培育高层次经济鉴证和服务类中介机构。新建社区服务网点100个，扶持社区服务企业50家。推进景区及各项旅游基础设施建设，创建4A级旅游景区1家、3A级景区2家，力争旅游人数、国内旅游收入均增长20%，外汇收入100万美元。大力促进电子商务、现代远程教育、网上医疗等信息服务业的发展，新发展固定电话12万部、互联网宽带用户5万户。

　　加大重点物流项目建设力度，加快中原沥青原料仓储配送中心、南外环物流中心等续建项目实施。开工建设南二环化工大市场、电子商贸城、豫北汽车城、香江百年商贸物流和生产资料集中采购中心。积极筹划实施桥北新镇物流园区、种子物流配送、小店物流港和新菏铁路新乡站煤炭物流基地等项目。推进商贸物流业民营化，吸引国内外大型物流企业落户新乡，依托107国道、京珠高速、京广线和环城快速路，集公、铁联运于一体，不断完善商品集散、中转、配送和货运代理、信息服务、电子商务等服务功能。

　　（五）大力推进体制创新，增强经济发展的内生动力

　　积极推进国有资产监管体制改革。对具备条件的政府投资公司和企业集团公司进行授权经营，向国有重点企业派出监事会。建立国有资本重大项目跟踪问效制度，确保国有资产保值增值。培育产权交易市场，促进国有资本有序流动和优化配置。认真落实国家、省关于投融资体制改革的决定，保障企业投资主体地位和决策自主权，进一步规范完善项目核准制、备案制，简化审批手续。主动适应新的项目管理方式，超前规划、申报、储备项目，增强经济发展后劲。

　　实施国有企业改革攻坚。紧紧抓住产权制度改革和国有大中型企业主辅分离两个重点，通过引进战略投资者、吸引民间资本，发展混合所有制经济。做好新飞公司、白鹭集团资产重组工作，实现投资主体多元化。稳妥实施中原棉纺织厂等一批企业的破产工作，力争市属国有和集体企业年底基本完成改制任务，委（局）属企业改制面达80%，各县（市、区）属国有企业上半年改制任务全部完成。高度重视改制企业职工安置，基本实现国有企业下岗职工基本生活保障向失业保险并轨。

　　积极推进其他各项改革。大力培育资本市场，采取股票上市、债券发行、信托融资等方式，扩大直接融资。完善创业投资体系，扶持中小企业特别是高新技术企业发展，培育新的经济增长点。力争实现金龙铜业上市和白鹭化纤再融资，做好黄河林业、太行振动、环宇公司、科隆电器上市准备和飘安、新亚集团等企业债券发行工作。积极支持工行新乡市分行、农行新乡市分行改革，协助做好呆坏帐核销剥离工作。搞好市商业银行增资扩股，降低不良贷款率。推进农村信用社改革央行票据申请和一级法人组建工作，确保央行票据兑付。巩固农村税费改革成果。全面完成粮食流通体制改革任务。深化县乡机构改革，积极推进事业单位机构改革，健全全市机关事业单位机构编制审核制度。加快社区建设，完善管理体制，创建国家、省级示范社区。

　　（六）健全对外开放工作体系，加快发展开放型经济

　　落实各县（市、区）及商务局、驻外专业招商机构、高新技术开发区、小店工业区等招商引资目标责任制、全员聘任制、承诺上岗制和“五职”责任人制度，健全各县（市、区）发展开放型经济评价体系。强力实施“东引西进”，打造承接东部产业转移的“桥头堡”，支持企业开拓西部市场。开展空心村、砖瓦窑及工矿废弃地整治，关闭污染严重、资源浪费和工艺落后企业，盘活土地存量资源，“腾笼换鸟”，招商引资。优化出口企业和贸易结构，实现外经贸主体多元化。做好参加“5?18”福州海峡投资贸易交易会的组织工作。加强与上海松江区的联络，促进城市间经贸合作。力争新批外商投资企业30家，新增拥有进出口经营权企业50家。外派国（境）外劳务人员1000人。

　　（七）落实促进就业再就业各项政策，健全社会保障体系

　　进一步加强社区劳动保障平台建设，健全劳动保障信息网，增加再就业小额贷款发放，加强城乡就业技能培训、职业介绍和就业指导，建立健全专业化、制度化、社会化的就业服务体系，逐步营造规范的城乡统筹一体化劳动力市场大格局。安置4500名“4050”人员就业。免费培训复员转业军人、下岗失业人员和农村富余劳动力5.3万人。做好大中专毕业生就业安置工作。加大监察执法力度，强化劳动合同管理，建立农民工工资保障制度，切实维护劳动者合法权益。

　　坚持宽进宽出，开展企业账户分设参保、农民参保等试点工作，提高个体私营等非公有制企业参保率，扩大社会保险覆盖面。依法加大征缴清欠力度，确保国有企业离退休人员养老金按时足额发放，做好失业职工保险金发放工作。进一步完善城市低保和农村特困户、五保户救助制度，健全城乡救助体系，探索建立农村社保体系。积极开展医疗、教育救助和法律援助工作。

　　（八）加强财政预算和税收征管工作，有效促进经济发展

　　严格财政纪律，强化部门预算、零基预算、国库集中收付、政府采购和“收支两条线”管理，认真执行人员编制与财政预算管理体制相结合的管理办法。合理调整财政支出结构，严格控制一般性支出增长，杜绝临时预算支出，保证法定和重点支出需要。完善市县乡三级财政管理体制，推进“乡财县管”，落实“三奖一补”政策，改善乡村财政状况。依法加强税收征管，深入开展纳税评估，强化税源监控和分类管理，充分挖掘税源及非税收入，严厉打击虚开增值税发票等涉税违法犯罪活动，防止关联企业域外逃税，保持税收稳定较快增长。

　　（九）坚持以人为本，统筹发展各项社会事业

　　认真做好“十一五”规划编制工作，高质量完成总体规划、专项规划、行业规划和县（市、区）规划编制，争取尽可能多的经济和社会事业发展项目进入国家、省规划。加强对经济社会发展质量的综合分析评价，促进全面、协调、可持续发展。

　　积极推进教育改革和发展。着力提高基础教育质量，整合优化教育资源，加快农村中小学布局调整，推进厂办学校移交政府管理。拓宽教育投资渠道，吸引社会资金办教育。加大城镇教师到农村对口支教工作力度。重点抓好市一中新校区建设、市外国语学校迁建和新乡职业技术学院组建工作。继续支持驻新高校加快发展，力争平原大学、新乡师专、教育学院合并升本成功。落实“人才强市”战略，注重培养、吸引、用好人才，重点吸引高层次人才和紧缺人才，搞好党政、企业经营管理和专业技术人才队伍建设。建立与全省联网的网上人才市场。

　　大力促进科技创新。搞好制造业信息化示范城市建设。争取省级以上科技计划项目30项。鼓励和帮助有条件的企业申报国家科技型中小企业技术创新基金。以重大产业科技攻关项目为纽带，促进高校、科研院所和企业建立战略联盟，双向共建研发中心。组织申报3－5项省高新技术产业化重点项目、3－5家高新技术企业、5－8项高新技术产品、100项以上专利。

　　做好人口、资源、环境工作。依法保护和合理开发利用国土资源，实行最严格的耕地保护制度。以水、大气污染防治为重点，强化环保执法和生态环境监管，加快推进造纸、水泥、采石企业结构调整和资源整合，抓好凤泉区周边采石、碎石等粉尘污染、东孟姜女河污染、海河流域和“南水北调”工程沿线污染治理及地下水保护等项目的实施，力争我市河流出境断面水质达标。坚决杜绝新增污染源，环评审批率和“三同时”执行率均达到100%。加快建立“依法管理、村（居）民自治、优质服务、政策推动、综合治理”的计划生育工作新机制，努力稳定低生育水平，提高出生人口素质。

　　推进文化、卫生等各项社会事业发展。加快文化产业发展，塑造牧野文化品牌，建设文化强市。做好平原博物院、新乡图书馆和档案市志馆的规划、选址、设计工作。编撰出版第二届《新乡市志》。促进新型农村合作医疗健康规范运作，建立健全突发公共卫生事件应急反应机制。完成第一次全国经济普查。加强国防教育，做好双拥共建和优抚安置工作。大力开展群众性体育活动。认真做好新闻出版、广播电视、防震减灾、民族宗教、人防、外事、侨务、气象等工作。

　　（十）发展社会主义政治文明和精神文明，努力营造良好的经济社会发展环境

　　坚持党的领导，自觉接受人大依法监督和政协民主监督，主动向人大及其常委会报告工作，向政协及其常委会通报情况。认真办理人大代表议案、建议和政协委员提案，不断提高办理质量。完善对市人大、政协检查、视察、评议的反馈制度，及时整改问题，不断改进和加强政府工作。广泛听取各民主党派、工商联、人民团体、无党派爱国人士意见，充分发挥工会、共青团、妇联等人民团体的桥梁纽带作用。巩固和扩大基层民主，完善村民自治、社区民主管理和企业民主管理，做好第五届村委会换届选举工作。

　　加强精神文明建设。坚持用邓小平理论和“三个代表”重要思想教育群众，广泛进行社会公德、职业道德和家庭美德教育，不断提高全社会思想道德水平。高度重视未成年人思想道德建设，积极在社区和新建住宅小区开辟未成年人思想道德教育阵地，营造有利于未成年人健康成长的社会环境。深入学习宣传我市先进群体，发挥典型示范作用。加强对外新闻宣传工作，提升新乡整体形象。

　　深化“五城”联创。以提高市民文明素质为核心，层层落实创建责任，健全完善城市管理长效机制，确保“中国优秀旅游城市”创建成功，力争完成国家园林城市、国家卫生城市创建任务，实现省级文明城市工作先进市目标。加强征信体系建设，建立健全社会信用制度、失信惩戒机制和信用信息共享机制，建立100家企业信息数据库，打造“信用新乡”。

　　继续整顿和规范市场经济秩序。依法打击制假售假、欺行霸市、商业欺诈、逃废银行债务等破坏经济秩序行为，切实抓好食品及药品专项整治。认真落实“企业安静工作日”、“无会月”和“首席服务官”制度，大力整治企业、学校周边环境，严肃查处“四乱”行为。高度重视安全生产工作，强化安全目标管理，继续抓好安全生产专项整治，积极推进基层基础安全工作，有效遏制重大事故发生。

　　切实维护社会稳定。深化 “全省综治先进市”创建活动，加强乡镇（街道）综治机构建设，完善群防群治网络，深入开展严打整治斗争。坚决打击敌对势力和“法轮功”等邪教组织活动。注重协调各方面利益关系，健全正确处理人民内部矛盾的工作机制，认真处理群众来信来访，主动化解各种社会矛盾，积极预防和妥善处置突发性群体事件，为人民群众创造安居乐业的社会环境。

三、加强责任、法治、服务型政府建设　全面提高行政水平

　　推进科学执政、民主执政、依法执政。制订《新乡市贯彻全面推进依法行政实施纲要的意见》，加强行政执法监督工作和体制建设。进一步减少行政审批事项，规范行政许可项目运作程序。加强行政决策机制建设，健全完善重大决策、重要行政许可事项听证公示制度和政府新闻发言人、公民旁听政府常务会议制度，完善政务信息网络，扩大发行《政府公报》，全面实行政务公开，充分保障人民群众知情权。

　　树立正确的政绩观。要时刻把人民的利益放在心上。牢记人民群众是公务员的衣食父母，关心群众疾苦、为人民谋利益是我们的责任和良知，要真正一心一意为群众办实事、办好事，决不允许借为民之名、谋个人升迁之实，坚决反对哗众取宠之举、浮躁作秀之风。要时刻把重视和关爱基层干部放在心上。更科学地做出工作安排，更客观地提出工作要求，设身处地改善他们的工作条件和待遇，满腔热情地引导他们提高工作能力和水平，保证和促进他们更好地干事创业。要时刻把实事求是放在心上。所有政府工作人员都要真正以振兴新乡为己任，多做打基础、谋长远的工作，不急功近利，不好大喜功，坚决杜绝“政绩工程”、“形象工程”。要时刻把“两个务必”放在心上。“尔奉尔禄，民脂民膏”，各级干部都要始终保持艰苦奋斗的作风，厉行节约，勤俭办事，反对享受摆阔、铺张浪费。建立财政预算执行工作领导责任制，加大对财政性资金使用结果和效益的审计、监察与评估、惩戒力度，有效降低行政成本，集中有限财力促发展、惠百姓、保稳定。

　　加强行政能力建设。深入贯彻十六届四中全会精神，全面提高公务员队伍的综合素质和行政水平，着力培育和提高各级干部驾驭社会主义市场经济、把握大局、加快发展的能力，增强工作预见性和超前性，善于应对各种复杂局面，处理好改革发展稳定的关系，为人民谋求更大福祉。以推进责任、法治、服务型政府建设为核心，严格实行行政首长问责制和行政效能监察制，建立健全一级对一级负责、一级支持一级的高效工作推进机制。始终坚持敢于创新、勇于负责，想干事、敢干事、会干事、干成事、不出事。始终坚持高标准、严要求，保持“不进则退、小进也是退”的忧患意识，增强只争朝夕、加快发展的紧迫感，以坚韧不拔的毅力、百折不挠的意志，迎难而上，奋力晋位升级。始终坚持不争权力大小、不争角色轻重、不争功过是非，做好卧薪尝胆、锲而不舍、长期奋斗的思想准备，埋头苦干，不辱使命。下决心转变政风，精简会议、文件，减少检查、评比，努力营造真抓实干、狠抓落实的工作氛围。强化监察、司法、审计、社会和舆论监督，从源头上预防和治理腐败，全力塑造廉洁勤政、务实高效、开拓创新的政府形象。

　　各位代表！新的一年，我们面临的任务艰巨而繁重，我们肩负的责任重大而光荣。让我们高举邓小平理论和“三个代表”重要思想伟大旗帜，紧密团结在以胡锦涛同志为总书记的党中央周围，同心同德，发奋图强，为把新乡建设成为中原城市群强市、实现中原崛起而努力奋斗！
